# Challenges for automatically extracting molecular interactions from full-text articles

**DOI:** 10.1186/1471-2105-10-311

**Published:** 2009-09-24

**Authors:** Tara McIntosh, James R Curran

**Affiliations:** 1 School of Information Technology, Faculty of Engineering and IT, University of Sydney, Sydney, Australia

## Abstract

**Background:**

The increasing availability of full-text biomedical articles will allow more biomedical knowledge to be extracted automatically with greater reliability. However, most Information Retrieval (IR) and Extraction (IE) tools currently process only abstracts. The lack of corpora has limited the development of tools that are capable of exploiting the knowledge in full-text articles. As a result, there has been little investigation into the advantages of full-text document structure, and the challenges developers will face in processing full-text articles.

**Results:**

We manually annotated passages from full-text articles that describe interactions summarised in a Molecular Interaction Map (MIM). Our corpus tracks the process of identifying facts to form the MIM summaries and captures any factual dependencies that must be resolved to extract the fact completely. For example, a fact in the results section may require a synonym defined in the introduction. The passages are also annotated with negated and coreference expressions that must be resolved.

We describe the guidelines for identifying relevant passages and possible dependencies. The corpus includes 2162 sentences from 78 full-text articles. Our corpus analysis demonstrates the necessity of full-text processing; identifies the article sections where interactions are most commonly stated; and quantifies the proportion of interaction statements requiring coherent dependencies. Further, it allows us to report on the relative importance of identifying synonyms and resolving negated expressions. We also experiment with an oracle sentence retrieval system using the corpus as a gold-standard evaluation set.

**Conclusion:**

We introduce the MIM corpus, a unique resource that maps interaction facts in a MIM to annotated passages within full-text articles. It is an invaluable case study providing guidance to developers of biomedical IR and IE systems, and can be used as a gold-standard evaluation set for full-text IR tasks.

## Background

Almost all known and postulated knowledge relating to biological processes is recorded in the form of semi-structured full-text articles. The volume of biomedical literature rapidly becoming available makes it very difficult for biologists to keep abreast of even their narrowest specialist fields. The traditional keyword-based Information Retrieval (IR) over abstracts often retrieves too many articles that must be individually inspected. To overcome this information bottleneck, there has been considerable interest in developing Natural Language Processing (NLP) tools to improve the accessibility of knowledge within articles [1,2]. In particular, there is a strong focus on the automatic extraction of interactions between bio-entities, such as genes and proteins [3-5].

The development and evaluation of effective NLP tools for the biomedical domain, requires new annotated corpora, as statistical models of language extracted from traditional newswire corpora are very inaccurate when applied to biomedical text. The most comprehensive annotated biological corpora available consist of sets of MEDLINE abstracts marked with linguistic information such as part-of-speech, anaphoric expressions, and syntactic structure, as well as biological annotation, marking entities such as proteins, genes and cells, and relationships between these entities [4,6-9]. As a results, most biomedical IR and IE systems, such as PubMed [10] and Medie [1], have been applied to abstracts only.

Unfortunately, the information in abstracts is dense but limited. For example, Friedman *et al*. [11] showed that only 7 out of 19 mentions of unique molecular interactions within a full-text article occur in the abstract. Full-text articles have the advantage of providing more information and repeating facts in different contexts across various sections, increasing the likelihood of an imperfect system identifying them. This redundancy can also be used for validating and ranking identified facts [12].

Full text contains explicit document structure, e.g. sections and captions, which can be exploited to improve IE. Regev *et al*. [13] developed the first biomedical IR system that specifically focused on limited text sections in full-text articles, such as figure captions. Their performance in the KDD Cup Challenge [14], showed the importance of considering document structure. Following Regev *et al*., others have investigated the importance of extracting information from specific sections. Yu *et al*. [15] retrieved synonyms of proteins and genes from abstracts and full text, and identified more synonyms with higher precision in full text, with the introduction section defining the majority of synonyms. Both Schuemie *et al*. [16] and Shal *et al*. [17] showed that the results and method sections are the most and least informative, respectively, for identifying gene mentions. In contrast, Sinclair and Webber [18] found the method sections useful in assigning Gene Ontology codes to articles.

These section specific results highlight the information loss resulting from restricting IR and IE to abstracts and other individual sections, as different sections often provide different information [16]. However, there has been little analysis of when the entire document is required for accurate knowledge extraction. For instance, retrieving a fact from the results section may require a synonym to be resolved that is only mentioned in the introduction. Despite this and the limited full-text annotated corpora available, IR competitions, like the Genomics track of TREC [19], require systems to retrieve and rank passages from biomedical full text that are relevant to question style queries.

In this paper, we explore the complexities biomedical IE systems will need to handle to exploit the knowledge contained throughout full-text articles. We investigated these difficulties by focusing on the manual identification of a set of facts about molecular interactions within full-text articles. The interaction facts we have annotated correspond to those identified and summarised in a *Molecular Interaction Map *(MIM) constructed by Kohn [20], and the articles in our corpus are those cited in each fact summary. The relevant passages form the foundation of our MIM corpus, and are manually annotated with linguistic phenomena, such as synonym use and anaphoric expressions.

One of the main issues for processing full-text which has not been addressed in other biomedical corpora, is understanding how text within one section, or even a single sentence, relies on other text within the same article to form a coherent argument. In this paper, we aim to identify how important this phenomena is for automatically extracting molecular interactions. We model this coherency in our corpus by not only identifying passages that directly state the interaction fact, but by including passages from which the fact can be inferred with the addition of knowledge detailed elsewhere in the document. We also annotate the passages containing this additional knowledge, which we call *dependencies*. These dependencies are not to be confused with syntactic dependencies. In our corpus, we identify synonym and extra fact dependencies. As a result, our corpus uniquely tracks the process of forming summaries of molecular interaction facts from full-text articles.

Our corpus also provides insight into the relative significance of other NLP tasks, such as the resolution of negated and coreference expressions. The negated expressions annotated in our corpus are not included in other corpora, and few biomedical corpora include coreference annotations [4,21,22]. These corpora only consist of abstracts or individual sentences, and thus do not reflect the level of importance of this task.

The MIM corpus also addresses a number of Information Retrieval (IR) issues, concerning the value of individual full-text sections and other document structures, such as figure headings, and fact redundancy. During the annotation process, we have documented the precise location that each fact and its dependencies were found. This leads to insights about the applicability of different sections for IE and IR. Additionally, we have exhaustively identified all mentions of the MIM facts in each cited article, allowing us to report on the level of fact redundancy and coverage within the articles. This also facilitates the use of this corpus as the first evaluation and analysis test set for a full-text biomedical sentence retrieval system. Using oracle experiments, we can quantify the performance upper bounds for keyword queries, and identify the importance of individual NLP components. We can also explore the characteristics of the resulting false positives and false negatives.

This paper is organised as follows: we first introduce the MIM and the process of identifying and annotating MIM facts. Through detailed examples, we then discuss the critical role of synonym and extra fact dependencies, followed by the guidelines for annotating negated and coreference expressions, and a presentation of our key findings from our corpus analysis. We then introduce the first use of the MIM corpus as a gold standard test and evaluation set for a sentence retrieval system, and provide a detailed performance analysis. The retrieval system and the evaluation metrics are the subject of the Methods section, at the end of the paper. The MIM corpus is available for research purposes and can be freely downloaded from 

## Results and Discussion

### Molecular Interaction Maps

**Figure 1:**
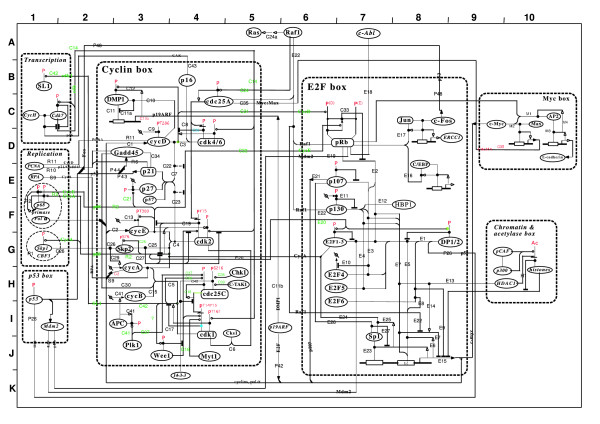
**Molecular Interaction Map**. The map corresponds to part A of the Molecular Interaction Map compiled by Kohn [20].

*Molecular Interaction Maps *(MIM) graphically depict the molecular interactions which occur between molecules of the same or different biochemical families, such as proteins, genes, amino acids, and multi-molecular complexes. Kohn [20] manually constructed a MIM based on scientific literature describing interactions in the mammalian cell nucleus, focusing on cell-cycle regulating molecules and the DNA repair process. Figure 1 shows the cell-cycle component of the MIM. This MIM includes 115 individual molecules (excluding complexes) and 203 interactions between them. The complete MIM diagram is shown in [20].

**Figure 2:**
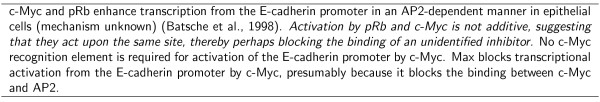
**MIM annotation M4**. Text composed by Kohn [20] which summarises the M4 interaction relationship depicted in Figure 1.

Each node in the MIM represents a molecule and the links between nodes correspond to interactions between the molecules. Each interaction link is assigned a unique key and is associated with a MIM description composed by Kohn [20] that summarises the evidence for the interaction from the literature, including citations. For example, Figure 2 contains the description passage for MIM interaction M4 (on the right of the Myc Box at grid reference C10 in Figure 1). It is important to note that the articles cited by Kohn for each MIM description are not exhaustive and thus many of the MIM interactions will be mentioned in other articles. The articles selected by Kohn however, present the primary research documenting the main interaction discoveries and/or findings. A tool capable of automatically extracting or augmenting a MIM would be extremely useful to biomedical researchers.

### Corpus annotation

In creating the MIM corpus we have attempted to reverse engineer the formation of Kohn's MIM descriptions by exhaustively tracing and documenting the process of identifying passages from the cited full-text articles that substantiate the MIM interactions. The MIM corpus consists of individual sentences or passages of text (referred to as *instances*) from the cited articles that the MIM descriptions can be inferred from. Each instance in our corpus is separately assigned the location within the article it was retrieved from and annotated for factual dependencies, and coreference and negated expressions.

#### Annotation process

The first stage in the development of the MIM corpus involved obtaining the full-text articles cited in the MIM descriptions. There are 262 articles cited by Kohn [20], and the MIM corpus currently consists of 2004 annotated passages from 78 full-text articles, supporting 76 MIM descriptions. An annotator with a biomedical background exhaustively identified these passages by manually reading each article several times. The corpus is restricted to the cited articles only. This allows us to quantify the need for external resources, e.g. synonym lists and ontologies. The annotation process involved the following:

1. For each MIM description, retrieve the full-text of the cited articles.

2. For each sentence in a MIM description, create a *main fact *to represent the knowledge conveyed.

3. For each main fact, identify and annotate each sentence or passage (*instance*) within the cited articles that the *main fact *can be inferred from. These include direct statements of the fact and passages that imply the fact. An instance of text is said to *support *the fact. We take a minimalist approach, and annotate the shortest sequence of text required to infer the fact, down to an individual sentence.

4. Main facts are often complex sentences, combining numerous facts from the cited articles. Passages from which part of a fact can be derived are also annotated. These instances are assigned to *subfacts *which are created to represent these partial facts. Each subfact must contain at least two of the bio-entities in the original main fact. Subfacts may also be broken down to represent less informative instances. The creation of subfacts (and subfacts within subfacts) is governed entirely by whether an instance is found that expresses part of the fact in question.

5. Many instances cannot be directly linked to their corresponding fact, as they *depend *on additional information from other passages within the full text or external domain knowledge. To represent these *dependencies*, new fact types are created — *synonym facts *and *extra facts*. All instances of these, within the same article, are annotated and a dependency link is added between the original instance and the new dependency fact. If an instance cannot be identified for a dependency fact, it is labelled as *undefined*.

6. Each instance is annotated with its location within the article, and linguistic phenomena, including negated and coreference expressions, which must be resolved for the MIM fact to be inferred.

#### Example corpus annotation

Consider the M4 MIM description in Figure 2. A main fact corresponding to the second sentence of this MIM description was created and is shown below:

Activation by pRb and c-Myc is not additive, suggesting they act upon the same site, thereby perhaps blocking the binding of an unidentified inhibitor.

**Figure 3:**
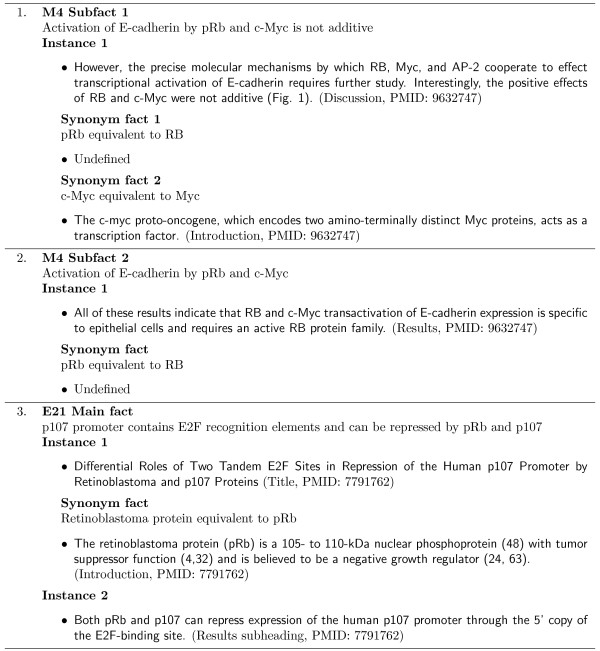
**Synonym fact examples**. Example MIM corpus instances which depend on synonym facts. Each instance is labelled with the article section it was located and its PMID.

Due to the complexity of the interaction relationships stated within this main fact, no single sentence or passage of text supporting this entire fact was identified within the cited article. However, instances of the M4 Subfact 1:

Activation of E-cadherin by pRb and c-Myc is not additive

and M4 Subfact 2:

Activation of E-cadherin by pRb and c-Myc

were identified, and thus these subfacts were created to represent this partial knowledge. Instances of these subfacts were located in the results and discussion sections of the cited article, and are shown in Examples 1 and 2 in Figure 3. Both of these instances depend on the resolution of synonyms to link the instances to the MIM description. Example 1, depends on two synonym facts. Synonym 1, states that the bio-entity *pRb *used by Kohn is equivalent to the bio-entity *RB *from the cited article, and no instance of this fact was identified in the article. Synonym 2 is required to map the term *c-Myc *to the synonymous term *Myc*, and an instance was identified in the introduction section of the cited article, as shown in Figure 3.

In order to create a corpus with as much coverage as possible for MIM main facts and subfacts, individual instances or parts of them may be associated with different facts. For example, the two consecutive sentences in Example 1 can only support M4 Subfact 1, however the first sentence also supports M4 Subfact 2. Thus the first sentence is also annotated as another instance of Subfact 2.

### Dependencies

A goal of our MIM corpus is to depict how the coherent flow of knowledge is presented or assumed within full-text articles. The dependency annotation is introduced when a main fact or subfact of a MIM description may not be entirely derived from the text of an instance alone. These instances depend on additional factual knowledge (*dependencies*), which may or may not be present in the same article, to allow the original MIM fact to be derived. In this section we discuss the two types of dependencies we annotate: synonym facts and extra facts.

#### Synonym facts

The frequent use of synonyms, metonyms, abbreviations and acronyms in biomedical text is a common source of ambiguity that is often hard for automated methods to resolve [23]. Furthermore, manually curated lists of these are difficult to maintain in rapidly moving fields like biology [24]. As a result there is considerable interest in developing systems to identify and extract these, e.g. [25-27]. However, there has been no investigation into the difficulties which arise from synonym use when automatically identifying cited facts from full-text articles.

In our corpus we group all synonyms, metonyms, and abbreviations, acronyms and other orthographic variations of bio-entities, excluding case changes, which need to be resolved to identify the original MIM fact, as *synonym facts*. For example, the synonyms (1) *E2F4*, (2) *E2F-4 *and (3) *E2F1-4 *in our corpus refer to the same entity *E2F4*, although the third term also includes the entities *E2F1*, *E2F2 *and *E2F3*. We do not include synonyms for other terms in the MIM fact, such as verbs.

In our first example in Figure 3, the instance which supports M4 Subfact 1 depends on two synonym facts. In the MIM description, the entity terms *pRb *and *c-Myc *are used, but in the relevant cited article (Batsche *et al*., 1998) only their synonymous terms, *RB *and *Myc*, are mentioned. Therefore there is a need for a synonym fact dependency.

To link the text in Instance 1 to the subfact, we must first identify that *pRb *is synonymously equivalent to *RB*, and form Synonym Fact 1 to represent this knowledge:

pRb is equivalent to RB

The next step is to identify all passages from within the same article which support this synonym fact. However, an instance was not identified and the synonym fact is labelled as *undefined*. This example highlights the ambiguity introduced when authors choose to use terms other than the ones within the articles they cite, and when synonymous terms are assumed to be general domain knowledge. In addition to this ambiguity, the term *RB *is also an abbreviation for *respiratory bronchiolitis*, *repetition blindness*, *ruminal buffer*, and *Rio bravo virus*, to name a few, and a homograph for the gene *ruby *(*rb*), *rabbit *(*rb*) and *rubidium *(*Rb*).

Instance 1 also depends on Synonym Fact 2:

c-Myc is equivalent to Myc

In this instance, the bio-entities *c-Myc *and *Myc *are used interchangeably, where the protein *Myc *is referred to by its gene name, *c-Myc*. The use of metonymy, where an entity can be substituted with another related entity, is common in biomedical literature, and an instance supporting this type of synonym fact was found in the introduction of the article. This synonym instance does not contain any common contextual patterns such as:

1. *X known as Y*

2. *X *(*Y*)

that are often used to extract sets of synonymous terms like *X *and *Y *[15,27]. Therefore, further processing to identify these synonyms via the causal relationship, *c-myc encodes Myc*, is required. After these synonymous terms are resolved, we can directly infer the MIM M4 Subfact from the instance.

Example 3 in Figure 3, shows two annotated instances of the MIM E21 Main fact, where only the first instance depends on a synonym fact to be resolved. The authors of the cited article used the long form of the bio-entity *pRb*, *Retinoblastoma Protein*, in the article's title (Instance 1). Thus to link the first instance to Kohn's MIM fact, these terms must be identified as synonymous. An instance supporting this synonym fact was identified in the introduction section of the article, stating the synonym fact clearly:

...*retinoblastoma protein (pRb)*...

After this statement, all later references to this protein in the article (excluding coreference expressions) used the shorter form, and thus Instance 2, identified in a result's subheading, does not depend on this synonym fact to infer the main fact.

#### Extra facts

Instances in the MIM corpus may also depend on extra information for the MIM fact to be inferred, which cannot be expressed by synonym fact dependencies. We created *extra fact *dependencies to represent and annotate this additional information need. Extra facts include all assertions (excluding synonym definitions) which are necessary to infer a main fact or subfact from an instance. Many extra facts are descriptions or classes of bio-entities, hyponym relationships and compounded terms. For example, in the extra fact below:

S465A-Abl is a mutated form of c-Abl where Serine 465 is substituted for Alanine

the bio-entity *S465A-Abl *is not a synonym of *c-Abl*, but a modified form of the protein.

**Figure 4:**
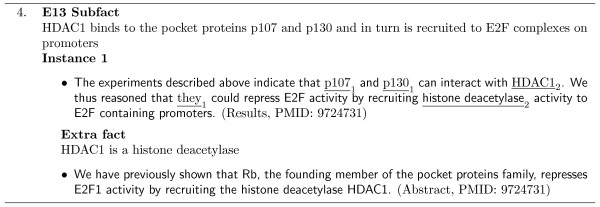
**Extra fact example**. An example MIM corpus instance which depends on an extra fact. Annotated anaphoric expressions are underlined. Note that the two different anaphoric expressions (1,2) are differentiated by their associated subscripts. The instance is labelled with the article section it was located and its PMID.

**Figure 5:**
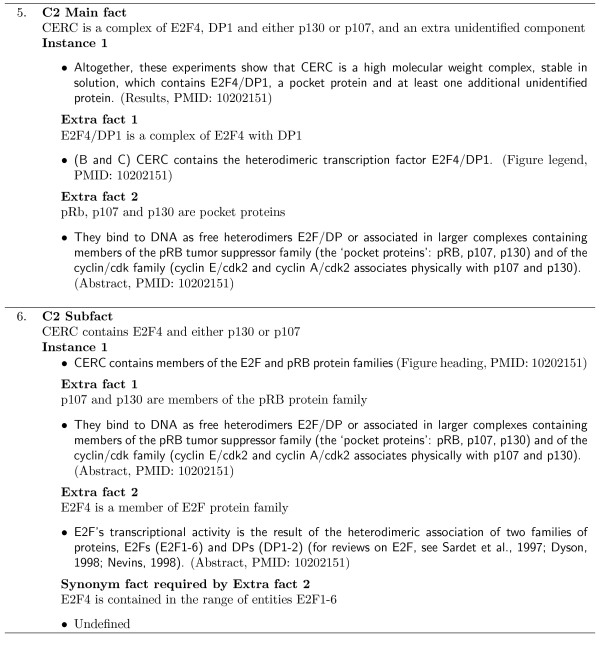
**Extra fact examples**. Example MIM corpus instances which depend on extra facts. Each instance is labelled with the article section it was located and its PMID.

If additional information is required for an instance to support a MIM fact, an extra fact is created. All supporting instances of these extra facts must then be identified within the same article as the original dependent instance. Examples of extra fact dependencies are shown in Figures 4 and 5.

Example 4, in Figure 4, shows an instance of the E13 Subfact that depends on the extra fact

HDAC1 is a histone deacetylase

to derive the subfact. The first sentence of this instance states the binding relationships between the bio-entities *HDAC1 *and *p107*, and *HDAC1 *and *p130*. This sentence does not support the entire subfact individually as the second sentence introduces the fourth required bio-entity, *E2F*. The extra fact is required to associate the class of proteins referred to in the second sentence using the term *histone deacetylase*, to the specific protein *HDAC1 *in sentence one. This is necessary as the sortal anaphor *they *in sentence two refers to the bio-entities *p107 *and *p130 *in sentence one, and not *HDAC1*. An instance supporting this extra fact was identified within the article's abstract, and is expressed in the apposition:

...*the histone deacetylase HDAC1*.

Once this extra fact is identified, the coreference expressions can be resolved, and in turn, the E13 Subfact can be inferred.

Two additional examples of extra fact dependencies are shown in Figure 5. Annotated instances of the C2 Main fact and one of its subfacts are shown. In Example 5, the instance supports the main fact only after two extra fact dependencies are established and resolved. These extra facts are required to map the bio-entity terms in the main fact to their corresponding terms/phrases in the instance.

The first extra fact represents the mapping between the bio-entities *E2F4 *and *E2F4/DP1*. It depicts a common representation of compounded bio-entities by using a slash (/) to represent a complex of multiple entities. This extra fact is identified in the instance by the following apposition:

...*the heterodimeric transcription factor E2F4/DP1*...

where the term *heterodimeric *states that *E2F4/DP1 *is a complex composed of two different proteins. Note that the slash notation is also often used to represent synonymous terms.

The main fact's instance also depends on additional factual knowledge to associate the coreference concept *a pocket protein *to the bio-entities *p130 *and *p107 *stated in the Main fact. To represent this dependency, another extra fact is created:

pRb, p107 and p130 are pocket proteins

and an instance defining this concept was identified within the article's abstract. The extra fact can be extracted from the expanded enumeration:

...*the 'pocket proteins': pRB, p107, p130*...

This extra fact's instance also details a hierarchy of bio-entity concepts where the *pocket proteins *are part of the concept *pRB tumor suppressor family*.

In Example 6, the instance of the C2 Subfact also depends on extra facts. The first extra fact is required to identify the bio-entities *p107 *and *p130 *in the subfact definition as members of the *pRB protein family *stated within the instance. The instance supporting this extra fact is the same text that supports Extra fact 2 of Example 5.

The second extra fact is necessary to associate the bio-entity *E2F4*, which is not mentioned in the subfact instance, as a member of the *E2F protein family *stated in the instance. An instance supporting this extra fact was identified, and defines the *E2F protein family *as:

...*E2Fs (E2F1-6)*...

This definition introduces additional complexity as the bio-entity *E2F4 *is not directly mentioned. The term *E2F1-6 *corresponds to multiple bio-entities, including *E2F4*. This information is represented in the corpus as a synonym fact, which defines *E2F1-6 *as a synonymous term for *E2F4*, and thus this extra fact instance also has a dependency fact.

#### Dependency graphs

Our corpus represents each of the main facts and subfacts as a dependency graph of instances, each which in turn may depend on other factual knowledge from synonym and extra facts. Each edge in the graph links an instance to each of its dependency instances. It is possible for an instance of a dependency fact to also depend on synonym and/or extra facts, as shown in Example 6 in Figure 5, where the instance of Extra fact 2 depends on a synonym fact. Thus paths of dependencies may occur, all of which would need to be resolved before the main fact or subfact could be derived from the initial instance.

### Linguistic phenomena

In the previous sections, we introduced the process of formulating main facts and subfacts from the MIM descriptions, and identifying supporting instances of these to annotate, along with any synonym or extra fact dependencies which they require. In this section, we will discuss the linguistic phenomena individual instances are annotated with. In our MIM corpus, we only annotated the linguistic constructs in individual instances that need to be resolved to infer a fact.

#### Negated expressions

The purpose of the negated expressions annotated in the MIM corpus is different to that of the BioScope corpus [28] and the BioInfer corpus [4]. In the BioScope corpus, negative terms in sentences, such as *not *and *neither*, and their scope are annotated for the purpose of developing systems which can detect uncertain facts or negative findings [28]. The BioInfer corpus is similarly annotated with negated expressions, however the annotated phrases correspond to those stating an absence of a relationship between entities, like *X not affected by Y *[4].

The negated expression annotation in the MIM corpus extend those in other corpora by focusing on statements that do not directly express a MIM fact, but from which the fact can be logically implied. Our annotations include logical negatives, as in the BioScope corpus, and lexical negatives which have not been annotated in either of the other corpora. Logical negatives are realised by a discrete, closed class negative particle like *not *or *no*. In lexical negatives, the negation is built into the lexical item, like *inhibit *or *mutant*. In these cases, the negated expression entails the opposite of a fact that would need to be worded differently.

As the MIM corpus is focused on molecular interactions, the main type of negated expressions identified correspond to statements describing modifications to molecules and their resulting effects. These statements document the outcomes of experiments from which one can identify/infer a molecule's function by modifying the molecule and observing any functional changes. For example, if in a gene knockout experiment we find that removing gene *X *results in function *Y *disappearing, we could infer that gene *X *is responsible in some way for function *Y*. In the literature, negated expressions are commonly used to describe these types of experiments, from which the normal function is inferred by the author and the reader. This typically requires two or more negated expressions to be processed simultaneously, as will be shown in the following examples.

**Figure 6:**
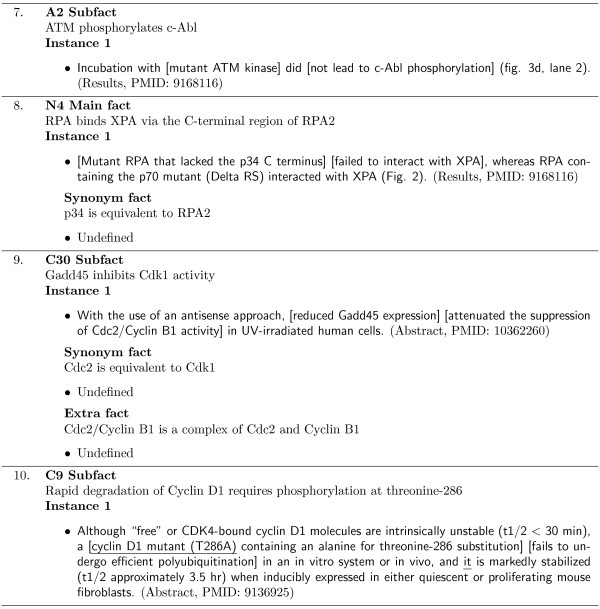
**Negated expression examples**. Example MIM corpus instances which are annotated with negated expressions. Negated expressions are marked with square brackets, and annotated anaphoric expressions are underlined. Each instance is labelled with the article section it was located and its PMID.

Figure 6 shows four example instances of different facts which require negated expressions to be interpreted for their corresponding MIM fact to be inferred. The negated expressions are marked by square brackets and, as in Vincze *et al*. [28], we annotate the full scope of the negated expressions.

In Example 7, two negated expressions within the instance need to be resolved to form the positive statement of the A2 Subfact. In this instance, the negated expressions are clearly defined. In the first negated expression, the lexical negated form of the bio-entity *ATM *is stated as *mutant ATM kinase*. The second negated expression is a logical negative which states the function the mutated form of *ATM *was unable to perform. Based on knowledge of the experimental aims and the implicit reporting of the results, one can logically combine these two negated expressions to infer the positive fact expressed in the A2 Subfact. If an IE system could not identify these negated expressions, then the incorrect relationship:

ATM does not phosphorylate c-Abl

may be extracted. It is these types of relationships that our MIM corpus aims to capture, with the goal of identifying processing errors like this.

The negated expressions annotated in Example 8, N4 Main fact, are similar in style to those in Example 7, however the processing to resolve the fact from this instance is more complicated. First, the synonymous terms *p34 *and *RPA2 *need to be resolved. Secondly, the first lexical negative has wider scope than in Example 7, as it states the specific type of mutation. This additional information is required to infer the main fact completely. As in Example 7, the first lexical negative expression is also followed by a logical negative expression, and these two negated expressions must be inverted and then combined to recover the main fact.

Negated expressions in the MIM corpus are not just identified by the presence of negated terms or lexical negative statements. For a negated expression to be annotated, the positive form of the expression needs to be resolved to infer its associated fact. For example, the instance of Example 8 also contains the lexical negative expression:

p70 mutant (Delta RS)

however as its resolution is not required for the corresponding MIM fact to be inferred, it is not annotated.

Many of the negated expressions annotated in the MIM corpus contain the terms *mutant *or *mutation*, which stem from the specific types of experimental studies performed in this domain. However, this is not always the case. Consider, for example, the subfact in Example 9 (Figure 6). The subfact description is itself a logical negative expression, and there is no reference to a mutated bio-entity in the supporting instance. The first logical negative expression, states the result of a different experimental technique (*an antisense approach*), which aims to silence or reduce the activity of the *Gadd45 *protein. And the second negated expression declares the result of this reduction, i.e. the *reduction *resulted in the *attenuation*. In the second negated expression, the nested negative phrase:

suppression of Cdc2/Cyclin B1 activity

correctly matches the action of *Gadd45 *stated in the MIM subfact, and it is thus not annotated. By inverting the two annotated negated expressions, we get the positive expression:

Gadd45 expression suppresses Cdc2/Cyclin B1 activity

and thus the MIM fact can be inferred from these negated expressions.

The last example in Figure 6 captures the complexity of negated expressions in the MIM corpus. The first negated expression is similar to the lexical negative expressions in the previous examples stating a mutation of *cyclin D1 at threonine-286 *directly. However, the second negated expression states that the mutated protein is unable to be *polyubiquinated*, which is not mentioned in the C9 Subfact. In turn, the inverted forms of these negated expressions do not directly convey the MIM subfact, and thus external domain knowledge is required.

In the first negated expression, we not only need to identify that the *threonine *required in the MIM fact is no longer a part of *cyclin D1*, but that the amino acid, *alanine*, it was substituted with cannot be *phosphorylated*. At this point, there is still no mention of *degradation*, however with domain knowledge this can be inferred from the second negated expression, as *polyubiquitination *of a protein triggers a signal for the protein to be degraded.

#### Coreference expressions

When automatically extracting information about a bio-entity, such as the interactions it is involved in, it is important to identify all textual references to that entity within the text, to ensure all information is retrieved. These textual references, for examples, *it, they *and *these*, are called coreference expressions. In biomedical literature, coreference expressions are frequently used to make abbreviated or indirect references to bio-entities or events.

**Figure 7:**
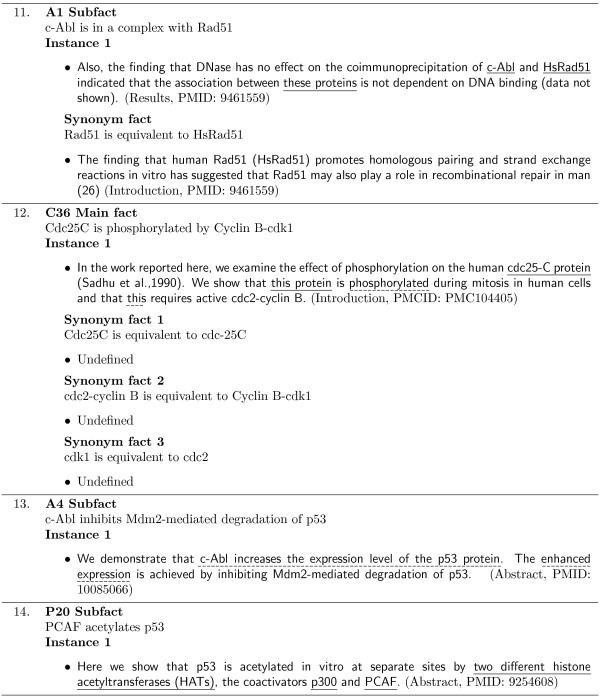
**Coreference expression examples**. Example MIM corpus instances which are annotated with coreference expressions. Anaphoric and cataphoric expressions are underlined, and event anaphora is marked with a dashed underline. Examples 11 and 12 contain anaphoric expressions, example 12 and 13 contain event anaphoric expressions, and example 14 contains a cataphoric expression. Each instance is labelled with the article section it was located and its PMID.

To quantify the importance of coreference expressions, instances in the MIM corpus are annotated with pronominal, sortal and event anaphoric expressions, and cataphoric expressions, including those referring to terms within another sentence. As in the negated expression annotations, only coreference expressions which need to be resolved to infer the MIM fact are annotated. Examples of annotated coreference expressions are shown in Example 4 (Figure 4), 10 (Figure 6), and 11-14 (Figure 7). The coreferring expressions and their referred terms are underlined with a single line.

In Example 11, the relationship of the MIM A1 Subfact is indirectly stated in the instance as:

...*association between these proteins*...

which can be expanded to:

...*association between c-Abl and HsRad51*...

which directly states the relationship. For an IE system to identify this relationship, the sortal anaphoric expression *these proteins *which is syntactically closer to the instance's relationship statement, would need to be linked to the proteins *c-Abl *and *HsRad51*.

A similar sortal expression appears in Example 4 (Figure 4), where the pronoun *they *in the second sentence refers to the proteins in the first sentence. However, this anaphoric expression is more complex to resolve. Firstly, the anaphor *they *does not specify what type of bio-entity it is referring to. Secondly, it is used to refer to only two of the three proteins (*p107 *and *p130*) in the first sentence. The third protein, *HDAC1*, is referred to in the second sentence with the anaphoric expression *histone deacetylase*. These anaphoric expressions need to be resolved, along with the dependencies, to link the information in both sentences together to form the MIM fact.

In the MIM corpus, we distinguish between the anaphoric expressions which refer to single or multiple entities, we have just described, from those that refer to events such as molecular processes. The MIM corpus *event anaphora *annotations differ to those described by Humphreys *et al*. [29], who link different sequential events together. The MIM corpus annotations provide links between references to the same events when their resolution is required to identify the MIM relationships.

Two examples of our event anaphora annotation are shown in Figure 7. Event anaphoric expressions are underlined with dashed lines. Example 12, is complicated as it not only contains an event anaphoric expression, but it contains two *this *terms. The first *this *is guided by the restricting modifier *protein*, and refers to the protein *cdc25-C *in the first sentence. The second *this *is the event anaphoric expression which refers to the phosphorylation event, *phosphorylated*. These two anaphoric expressions help resolve the MIM fact by linking the bio-entities in each sentence to the phosphorylation relation described.

In Example 13, the event statement in the first sentence indirectly details part of the relationship described in the A4 Subfact. It is in the second sentence, that the specific MIM fact relationship is described, however the event anaphoric expression, *the enhanced expression*, needs to be resolved first. This event anaphor links the two interaction relationships together, which in turn introduces the bio-entity *c-Abl *in the first relationship to be syntactically associated with the second relationship (*inhibiting Mdm2-mediated degradation of p53*).

In the MIM corpus, cataphoric coreference expressions within instances, which need to be resolved to infer their associated MIM facts are also annotated. Cataphoric expressions, like anaphoric expressions, are textual references, however they refer to bio-entities which are detailed in the text after the coreferring expression. An example of this linguistic phenomena is shown in Example 14 in Figure 7. In Example 14, to identify the *acetylating *relationship between the bio-entities *PCAF *and *p53*, where *PCAF *is syntactically distant from the relationship statement, the cataphoric expression:

two different histone acetyltransferases (HATs)

needs to resolved. This expression refers to the following bio-entities *p300 *and *PCAF*. As this cataphoric expression is syntactically closer to the relationship statement, if it is resolved, the relationship between the bio-entities, *p53 *and *PCAF *(as well as *p53 *and *p300*), can be recovered.

### Corpus analysis

Our MIM corpus is the first of its kind to explore the complexity of full-text articles with a focus on the development of IE and IR systems. Our analysis provides an overview of the tasks involved and difficulties which may arise when extracting knowledge from full text.

The MIM corpus consists of text segments taken from 78 full-text articles used as references by Kohn [20]. In total, we have identified and annotated 2162 sentences from these articles, which document the facts contained in 76 MIM summaries of molecular interactions by Kohn. Table 1 shows the distribution of the various fact types which have supporting instances identified and annotated. To reverse engineer the knowledge presented in the 76 summaries, we constructed 134 different main facts. Of these, 107 main facts had supporting text identified in their corresponding articles. We identified and annotated 363 different instances of text which support these main facts. The 27 unidentified main facts stated complex information that was not expressed within a single passage of text. Each of these main facts are however supported by instances of subfacts that express part of the knowledge they convey. Note that since subfacts were only created when instances supporting part of a fact or subfact are identified, all subfacts have supporting instances (247 of 247) by definition. There are a total of 729 different facts created, including 135 synonym facts and 213 extra facts, with only 67% (492) of these facts having instances identified. This low percentage primarily results from identifying only 39 of the synonym facts required. The proportion of missing synonym and extra facts shows the importance of creating external resources, such as ontologies, and tools for recognising orthographical variants, for the use of IE and IR systems.

**Table 1:** Fact types in the MIM corpus

**Fact type**	**No. Created**	**No. Identified**	**No. Instances**
Main fact	134	107	363
Subfact	247	247	1468
Synonym fact	135	39	48
Extra fact	213	99	125

Total facts	729	492	2004

#### Fact redundancy

Unlike other biomedical corpora available, in the MIM corpus we have annotated interaction facts which are repeated in an article, often in different contexts. This is a direct result of our annotation effort not been restricted to abstracts or single sentences, which are limited in space and thus the information they can convey. Using full-text articles which tend to repeat the main findings numerous times, we are able to annotate all instances of individual facts. As a result, the MIM corpus has a high level of fact redundancy, and this type of redundancy can be incorporated into systems to improve the extraction process. For example, Clarke *et al*. [12] showed that redundancy can be exploited by Question Answering Systems by aiding the passage selection components, as retrieved potential answers with high redundancy within documents are often more correct than others. Imperfect systems can also benefit from fact redundancy, as the chances of extracting a fact repeated in different contexts increases. In the MIM corpus, the most redundancy occurs in main facts and subfacts, with on average 3.4 and 5.9 instances each respectively, while the synonym and extra facts have almost no redundancy.

#### Dependencies

Table 2 shows the percentage of instances which depend on synonym and extra facts in our corpus. In total, 76.9% of main fact instances have at least one dependency, with 54.0% and 35.0% depending on at least one synonym fact or extra fact, respectively. However, only 10.2% of main fact instances which depend on a synonym fact have it defined within the same article. These main facts are considered to be completely contained within the cited article, requiring no external resources to resolve them. Many subfact instances also depend on synonym and extra facts, however fewer of these instances, in particular those depending on synonym facts, are completely contained within the articles — a direct result of the small number of synonym facts created which had a supporting instance identified. Interestingly, some synonym and extra facts depended on other synonym and extra facts, where the majority of these additional dependencies were undefined.

**Table 2:** Instances with dependencies.

**Instance type**	**Total dependencies**	**Synonym fact**		**Extra fact**	
Main fact	76.9	54.0	(10.2)	35.0	(19.6)
Subfact	57.5	34.8	(4.4)	31.9	(14.9)
Synonym fact	10.4	6.2	(2.1)	4.2	(0.0)
Extra fact	19.2	13.6	(0.0)	6.4	(5.6)

Percentage of instances in the MIM corpus which have at least one synonym and/or extra fact dependency (total dependencies). The percentage of instances which depend on at least one synonym and extra fact are also shown. The number in parentheses corresponds to the percentage of instances for which an instance of the dependent fact was identified.

Our corpus contains more synonym than extra fact dependencies (Table 2), however there are more unique extra facts and more instances of these identified in the articles (Table 1). A large proportion of main fact and subfact instances have dependencies (Table 2). Since only a small percentage of these dependencies are identified, many of these main facts and subfacts are not completely contained within the articles. This further demonstrates the importance of automatically extracting resources for these dependency facts.

As seen in the annotation examples, a single instance can depend on multiple synonym and extra facts for the original MIM fact to be inferred. For a given instance, we refer to the number of these dependencies spanning from the instance as its *dependency breadth*. Table 3 shows the degree of dependency breadth for the instances within the corpus. In total, 277 of the main fact instances and 842 of the subfact instances have one or more dependencies. Many instances of main facts (44.1%) and subfacts (37.8%) depend on only one fact.

**Table 3:** Breadth of instance dependencies.

**Breadth**	**Main fact**		**Subfact**		**Synonym fact**		**Extra fact**	
1	160	(44.1)	554	(37.8)	3	(6.2)	19	(15.2)
2	99	(27.3)	246	(16.8)	1	(2.1)	4	(3.2)
3	16	(4.4)	41	(2.8)	0	(0.0)	0	(0.0)
4	1	(0.3)	1	(0.1)	0	(0.0)	0	(0.0)
6	1	(0.3)	0	(0.0)	0	(0.0)	0	(0.0)

Number of instances in the MIM corpus depending on multiple facts. The number in parentheses corresponds to the percentage of instances.

As many instances depend on other facts, it is fortunate that most of the instances depend on less than three different facts. This is because each additional dependency will reduce the likelihood of an instance being identified by an automated system. However, considering that the instances and their dependency facts may occur anywhere within an article, automatically extracting them is still a very challenging task.

In the MIM corpus, an instance of a dependency fact may also depend on synonym or extra facts. We call these *dependency chains*. The facts within a dependency chain must all be resolved before the original fact can be inferred. An example of a dependency chain is shown in Example 6. For a given instance, the maximum length of the dependency chain is referred to as its *dependency depth*, and instances with one dependency have a dependency depth of 1.

Table 4 shows the distribution of the dependency depths spanning from instances of each fact type. The majority of main fact (66.9%) and subfact instances (51.9%), have a dependency depth of one. This means that very few instances of dependency facts also rely on additional dependencies — only 34 main fact instances and 75 subfact instances require a chain of two dependencies to be resolved. This distribution is also fortunate, as the introduction of dependency chains is likely to significantly impair an IE system's performance.

**Table 4:** Depth of instance dependencies.

**Depth**	**Main fact**		**Subfact**		**Synonym fact**		**Extra fact**	
1	243	(66.9)	762	(51.9)	4	(8.3)	22	(17.6)
2	34	(9.3)	75	(5.1)	0	(0.0)	1	(0.8)
3	0	(0.0)	5	(0.3)	0	(0.0)	0	(0.0)

Number of dependency chains in the MIM corpus. The number in parentheses corresponds to the percentage of instances.

#### Locating facts

Each instance in the MIM corpus is also annotated with its location within the cited article. These include specific article sections, such as abstracts and conclusions, as well as other structures, like the article's title, or headings and captions. Using this data, we can evaluate the informativeness of each section and structure for identifying molecular interactions and specific fact types. By incorporating our detailed dependency annotations, we can also determine how many instances depend on additional facts which have instances defined in different sections. This allows us to evaluate the relative importance of processing each article as a complete discourse for fact extraction.

Table 5 shows the percentage of facts which have at least one supporting instance identified within particular sections of the articles. The number in parentheses corresponds to the percentage of instances which are completely contained within a section, that is, the instance and all of its dependencies are identified in the same section. Note that as each fact may have multiple instances which may be identified in different sections, the percentages do not sum to 100.

**Table 5:** Locations of facts in full-text sections.

**Article section**	**Main fact**		**Subfact**		**Synonym fact**		**Extra fact**	
Title	8.2	(1.5)	8.9	(3.2)	0.0	(0.0)	0.0	(0.0)
Abstract	42.5	(18.7)	38.1	(23.9)	13.3	(13.3)	14.6	(13.1)
Introduction	20.9	(6.7)	32.8	(18.6)	13.3	(12.6)	9.9	(8.0)
Results	43.3	(20.9)	73.7	(35.2)	3.0	(3.0)	16.9	(13.1)
Discussion	36.6	(14.9)	44.5	(21.1)	0.7	(0.0)	3.8	(3.8)
Figure Heading	9.7	(1.5)	30.8	(13.4)	0.7	(0.7)	0.0	(0.0)
Figure Legend	7.5	(0.7)	16.2	(9.7)	0.0	(0.0)	5.2	(5.2)
Table Data	0.0	(0.0)	0.4	(0.0)	0.0	(0.0)	0.0	(0.0)
Methods	0.7	(0.0)	2.0	(0.8)	0.0	(0.0)	4.2	(3.3)
Conclusion	1.5	(0.7)	1.6	(1.2)	0.0	(0.0)	0.0	(0.0)
Footnotes	0.0	(0.0)	0.0	(0.0)	3.7	(3.0)	0.0	(0.0)
Section Headings	11.2	(1.5)	22.3	(9.7)	0.7	(0.7)	0.5	(0.5)

Percentage of facts which have at least one instance identified in a specific article section. The number in parentheses corresponds to the percentage of facts for which all of their dependencies are identified within the same section.

Many of the main facts are identified in the results and abstracts of articles, however these individual sections account for less than 45% of the main facts with instances annotated. More than 70% of subfacts had a substantiating instance within the results section, whereas only 38.1% were identified within abstracts. This provides a clear indication that systems which process only abstracts will be disadvantaged by the significant information loss.

The best sections for identifying synonym facts were the abstract and introduction sections, with few being identified within other locations. This is not too surprising, as it is more appropriate to introduce abbreviations and other synonyms when they are first used within an article. This finding is similar to that identified by Yu *et al*. [15].

The majority of extra facts were located in the results and abstract sections. Interestingly, the conclusion and methods sections of the articles rarely contributed any facts. The conclusion sections predominately discussed speculative ideas and future directions, while the methods sections detailed experimental procedures and conditions. Surprisingly, due to their restricted length, we also found the section and figure headings to express many subfacts.

Our location analysis so far indicates the usefulness of each section for expressing different fact types, however it does not consider the degree of instance redundancy which can be exploited by systems. More specifically, Table 5 only considers if a fact type can be identified within a section, not whether it appears multiple times.

Table 6 shows the percentage of instances which are identified in particular article sections, and indicates the level of redundancy within sections. For example, a fact may have multiple instances within the results section, which is referred to as instance redundancy. For synonym facts, which often have no instance redundancy, the abstracts and introductions are still the most useful sections for identifying them. Not too surprisingly, the section and figure headings do not express many instances as they are limited in both number and length.

**Table 6:** Locations of instances in full-text sections.

**Article section**	**Main fact**		**Subfact**		**Synonym fact**		**Extra fact**	
Title	3.9	(0.6)	1.5	(0.5)	0.0	(0.0)	0.0	(0.0)
Abstract	19.0	(7.7)	9.5	(5.9)	37.5	(37.5)	26.4	(24.0)
Introduction	9.1	(2.5)	9.5	(5.1)	37.5	(35.4)	17.6	(14.4)
Results	30.3	(11.3)	39.4	(17.7)	8.3	(8.3)	34.4	(25.6)
Discussion	24.8	(8.3)	19.1	(8.4)	2.1	(0.0)	6.4	(6.4)
Figure Heading	4.4	(0.6)	8.9	(4.6)	2.1	(2.1)	0.0	(0.0)
Figure Legend	2.8	(0.3)	5.4	(3.3)	0.0	(0.0)	8.8	(8.8)
Table Data	0.0	(0.0)	0.1	(0.0)	0.0	(0.0)	0.0	(0.0)
Methods	0.3	(0.0)	0.3	(0.1)	0.0	(0.0)	7.2	(5.6)
Conclusion	0.6	(0.3)	0.3	(0.2)	0.0	(0.0)	0.0	(0.0)
Footnotes	0.0	(0.0)	0.0	(0.0)	10.4	(8.3)	0.0	(0.0)
Section headings	5.0	(0.6)	6.0	(3.1)	2.1	(2.1)	0.8	(0.8)

Entire article	100.0	(32.0)	100.0	(49.0)	100.0	(93.8)	100.0	(85.6)

Percentage of instances which are identified in specific article sections. The number in parentheses corresponds to the percentage of instances for which all of their dependencies are identified within the same section.

The best sections for finding repeated instances of main facts and subfacts are the results and discussion sections. This contrasts with the results in Table 5, where the abstracts were shown to have a significant number of facts identified. This difference is due to the different lengths of these sections and their purposes. For example, abstracts are limited in size and thus the facts they present are predominately only stated once. Therefore, an IE system restricted to only abstracts must cover all possible ways a molecular interaction can be stated to ensure its extraction, as the system cannot rely on redundancy for validation or for catching a missed fact in another context later.

For the 2002 KDD Cup Challenge, Regev *et al*. [13] developed an IR system which specifically focused on limited text sections, such as titles and figure headings. In the MIM corpus, these sections are poorly represented, however when they do state an interaction fact they do so very concisely.

In our next analysis we consider the dependencies of different fact types and their instances. If an IE system is restricted to a particular section, as they often are to abstracts, all instance dependencies must also be identified within the same section. When we take into account each instance's dependencies the results for each section drop dramatically (those in parentheses in Table 5 and 6). For example, main fact instances which are predominately expressed in the results section, decreases from 30.3 to 11.3% (Table 6).

That is, only 11.3% of main fact instances could be completely recovered within the results section alone. This is a direct result of the synonym and extra fact dependencies that are mainly defined in the abstract and introduction sections, and are thus not identified in the results section with the dependent instances. This is similarly observed with instances in the abstract section, where the majority of dependency facts are defined elsewhere. These results further demonstrate the need for processing an article as one discourse, rather than as individual disjoint sections, to allow the resolution of synonyms and extra facts stated in different sections, while gaining redundancy coverage from the results and discussion sections.

#### Negated and coreference expressions

Table 7 shows the percentage of instances annotated with negated and coreference expressions in the MIM corpus. We have separated the coreference expressions into three main groups: pronominal and sortal anaphora (anaphora), event anaphora, and cataphora. Each of the individual annotated expressions appear in less than 10% of the instances, with standard anaphoric and negated expressions the most predominate. Very few instances are annotated with cataphoric expressions (2.0%).

**Table 7:** Annotated expressions.

**Expressions**	**Instances**
Negated	5.5
Coreference	13.0

Anaphora	9.4
Event Anaphora	2.6
Cataphora	2.0

Percentage of instances which are annotated with linguistic expressions.

Negated expressions are the second most common linguistic property annotated following standard anaphoric expressions. These expressions appear in 5.5% of instances, and pose an interesting NLP task for the automatic extraction of these molecular interaction relationships. The identification of mutation mentions has been investigated by Caporaso *et al*. [30] and Erdogmus *et al*. [31], however the cause and effect of mutations with respect to molecular interactions has not been investigated.

In total, 13% of the instances are annotated with coreference expressions, which are necessary to identify their corresponding facts. However this is only a subset of those appearing in the MIM corpus. In fact, 20% of the instances contain at least one of the following coreferring terms: *these*, *These*, *they *and *They*. Although less than 15% of instances require an anaphoric expression to be resolved, an IE system with an anaphora resolution component, must attempt to resolve all anaphoric expressions as it is not known in advance which expressions need resolving. These results suggest that we would expect the greatest improvement when systems incorporate anaphora resolution components, and little improvement from cataphoric expression analysis.

### Application

Most biomedical corpora, such as GENIA [6-8] and BioInfer [4], have been annotated for the purpose of aiding the development of specific IE systems. The GENIA corpus was recently the focus of the BioNLP'09 Shared Task on Event Extraction [32]. One of the main objectives was to identify biological events in text marked with bio-entities. The state-of-the-art performance of 51.95% (F-score) was achieved using a combination of sophisticated NLP methods such as parsing, Support Vector Machines with graph-based features, and rule-based techniques [33].

In this section, we consider the related task of identifying sentences and passages that are likely to contain scientific results. We present the first use of the MIM corpus as a gold standard evaluation dataset for a full-text sentence retrieval system. We perform oracle experiments to estimate the performance upper bound for different types of keyword queries, and to investigate how much improvement could be achieved if systems can accurately process linguistic phenomena like anaphora, negation and hedging.

The task of the retrieval system is to identify sentences from the cited full-text articles which report relationships documented in the MIM facts. The system is restricted to the articles in the MIM IR corpus, which consists of those available in HTML format. The system, data and performance measures used, are described in the Methods section.

**Figure 8:**
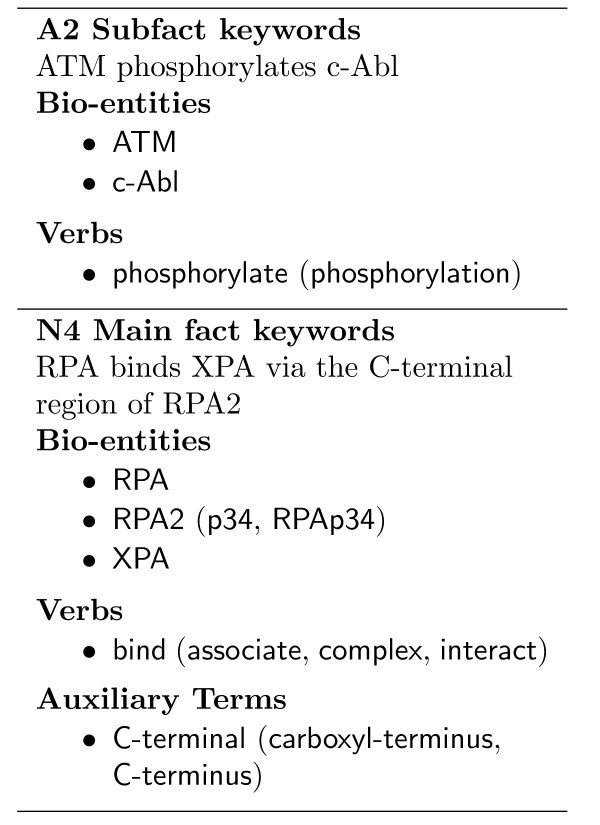
**Query keyword set examples**. Example query keywords for the A2 Subfact and the N4 Main fact in Figure 6. Example synonyms of keywords are shown in parentheses.

Since we have exhaustively identified and annotated all of the sentences supporting these specific facts, we can use the MIM IR corpus as a gold standard dataset to reliably identify all relevant and irrelevant retrieved sentences and report on the accuracy of the system. Each of the main facts and subfacts identified in the MIM IR corpus are assigned a set of keywords. Figure 8 shows two example sets of keywords. The keywords include the bio-entities and their synonyms, involved in the facts associated molecular interaction, and the verbs which describe the interaction. An additional set of terms referred to as the *auxiliary *terms are also included. Using these keyword sets, different queries for the IR systems are constructed. This is explained in further detail in the Methods section.

#### System performance

The overall performance of the sentence retrieval system using as input the different query sets for each of the MIM facts is shown in Table 8. Table 8 shows the number of sentences retrieved by each query set, and the precision (*P*), recall (*R*) and F-score (*F*), and the distribution of true positive (TP), false positive (FP) and false negative (FN) sentences of each query.

**Table 8:** Sentence retrieval performance.

**Query set**	**No. retrieved**	**P**	**R**	**F**	**TP**	**FP**	**FN**
bio-ent + verb + auxiliary	1769	34.0	34.6	34.3	601	1168	1135
bio-ent + verb	2287	28.3	37.3	32.2	647	1640	1089
bio-ent + verb syn + auxiliary	3277	33.2	62.7	43.4	1089	2188	647
bio-ent + verb syn	4507	25.8	67.1	37.3	1165	3342	571
bio-ent + any verb	8856	14.4	73.4	24.1	1274	7582	462
bio-ent	10232	12.7	74.9	21.7	1300	8932	436

Effectiveness of query sets on sentence retrieval performance. For each query set, we identified the number of sentences retrieved, and the precision, recall, and F-score. The number of true and false positives, and false negatives are also shown.

The first experiment in Table 8 corresponds to the most restrictive query set, requiring all of the keywords for a MIM fact: bio-entities, main verbs, and auxiliary terms, to be present in the retrieved sentences. This query is unrealistic because it requires a user's knowledge of the exact relationship stated in each of the MIM facts, including the specific verbs and auxiliary terms used. As a result, this query composition identifies the least number of sentences, and achieves the highest precision of 34.0%, but the lowest recall of 34.6% (65% of the sentences annotated in the MIM IR corpus are not identified, with 1135 FN).

Each subsequent experiment shown in Table 8 relaxes the search criteria, for example by expanding the verb query set to include synonymous terms. This increases the number of sentences retrieved and recall, however there is the expected or usual decrease in precision. The least restrictive search, ent, results in the largest recall and the lowest precision, and returns an enormous number of FP sentences.

There is an improvement in F-score, from 32.2 to 37.3%, when the corresponding verb lists are expanded to include their synonyms (bio-ent + verb syn). There are approximately 50% less FN, however the number of FP increases by a similar percentage. The best performance of 43.4% F-score is achieved with the bio-ent + verb syn + auxiliary query set. The search restriction enforced by the auxiliary terms reduces the number of FP by 34%, however including these terms unrealistically models a user's search style as it relies heavily on prior knowledge of the exact MIM fact. Unfortunately, the most realistic query setting is ent + any verb, since it is feasible to enumerate possible interaction verbs without prior knowledge of the specific type of interaction.

#### Characteristics of false negatives

**Figure 9:**
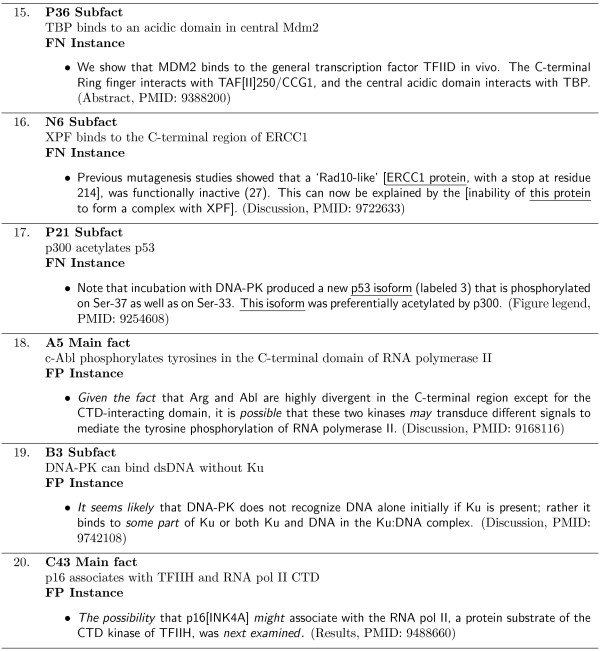
**False negative and false positive examples**. Examples of false negative instances are shown in examples 15-17, and false positive sentences are shown in examples 18-20. Hedging and commitment statements are italicised. Negated expressions are marked with square brackets, and annotated anaphoric expressions are underlined. Each instance is labelled with the article section it was located and its PMID.

We were interested in understanding why some of the annotated instances are not retrieved by the system, that is the false negatives (FN). Using the MIM corpus, we can determine the linguistic phenomena or dependencies of each FN which may be responsible for them going undetected. This enables us to evaluate the potential impact these phenomena have on the sentence retrieval task and potentially other NLP tasks. Examples 15-17, in Figure 9, are FN instances of their respective facts.

Table 9 shows the percentage of FN instances containing different types of linguistics phenomena. Note that as the bio-ent query contains all synonyms, no FN will arise due to lack of synonym knowledge. In all experiments, the majority of the FN instances can be linked to some form of linguistic phenomena. Only 12.1% of the FN resulting from the most relaxed search (*bio-ent*) do not have any linguistic annotations or extra fact dependencies. For example, Instance 15 in Figure 9, consists of two consecutive sentences without any coreference expressions. Few FN need negated or cataphoric expressions to be resolved to identify the sentences. These are however, required to extract the relationship between the bio-entities once the sentences are identified.

**Table 9:** Annotated phenomena in false negative instances.

**Query set**	**Negated**	**Anaphora**	**Event ana.**	**Cataphora**	**Extra fact dep.**	**None**
bio-ent + verb + auxiliary	6.3	10.0	3.2	1.1	37.5	30.5
bio-ent + verb	6.6	9.9	3.3	1.1	37.0	30.7
bio-ent + verb syn + auxiliary	5.9	14.3	5.6	1.4	48.5	18.0
bio-ent + verb syn	6.5	14.9	6.5	1.6	47.6	16.8
bio-ent + any verb	6.7	17.2	7.4	2.0	46.5	14.5
bio-ent	6.6	17.6	7.7	2.2	48.9	12.1

Percentage of linguistic phenomena annotated in the false negative instances for each query set.

The main linguistic phenomena associated with the FN instances are the anaphoric expressions and extra fact dependencies. For example, 17.6% of FN instances resulting from the bio-ent query contain an anaphoric expression, and 48.9% require at least one extra fact dependency to be resolved. Example 16 and 17 show instances where no single sentence mentions each of the queried bio-entities, and an anaphoric expression is required to identify the MIM facts that are conveyed across multiple sentences. Therefore, an anaphora resolution system has the potential to not only improve the relationship extraction process, but also to improve the number of relevant sentences retrieved. Furthermore, the large proportion of FN depending on extra facts, even with the most restrictive search (37.5%), shows that there is a need for systems to identify these dependencies, and treat full-text documents as a complete discourse rather than a set of individual sentences.

#### Characteristics of false positives

Our next analysis focuses on the large numbers of FP sentences retrieved in the query experiments. Results in scientific literature contain various levels of certainty, ranging from speculation to complete confidence, and after manually inspecting a small subset of the FP, we identified a common use of hedging within the sentences. *Hedging *is frequently used in scientific literature to indicate any lack of commitment to a fact [34]. For example, the following sentences express the same proposition between two proteins *RPA1 *and *DNA-PK*, however only the first does so with certainty:

1. *RPA1 was sufficient to form a complex with DNA-PK*.

2. *These results suggest that RPA1 interacts directly with DNA-PK*.

In this section, we aim to identify any potential FP reduction that may be gained from recognising hedging and commitment in articles. More specifically, can hedging and/or commitment be used to help separate relevant and irrelevant sentences. Hedging has been studied in the citation analysis of scientific literature [35], and is also annotated within the BioScope corpus [28]. Our study is the first we are aware of which investigates the impact of hedging on biomedical sentence retrieval.

Hedging is typically realised using modal verbs, epistemic adjectives, nouns and adverbials, lexical verbs and indefinite quantifiers [36]. For our analyses reported here, we experimented with terms in each of these categories that express uncertainty, and the small set of speculative terms identified by Light *et al*. [37]. We also constructed a list of commitment terms, which express the reverse — a high degree of certainty. Examples of each of these lists are shown in the Methods section. Using these lists, we can compare the frequency of hedging and commitment terms within the TP and FP sentences.

Examples 18-20 in Figure 9 are FP of their respective facts and contain at least one hedging expression. In these instances, the authors are indicating their research aims and hypotheses, however this is only directly stated in Example 20, indicated by the phrase *was next examined*. In Example 18, the epistemic adjective *possible *and modal verb *may *are used to express open-mindedness about the MIM fact. This instance also expresses certainty, using the phrase *Given the fact*, but about another statement.

Epistemic adjectives and modal verbs are also used in Examples 19 and 20, respectively. If we ignore the notion of hedging, only Examples 18 and 19 would match the MIM facts. Although Example 20 contains all bio-entities and the exact verb used to describe their relationships (*associate*), it only substantiates part of the MIM fact (*p16 associates with RNA pol II*).

Table 10, shows the results of our hedging and commitment analyses on the output of the sentence retrieval system with the *bio-ent + verb syn *query sets. The majority of the hedging categories occur in less than 7% of TP, FN and FP, with little class discrimination. Epistemic lexical verbs and the speculative words identified by Light *et al*. [37] are the most frequently occurring hedging terms within the literature, however they are also not discriminative. The most significant class discrimination is identified with the modal verbs, with a high 15% of FP (corresponding to 246 FP) containing at least one modal verb. There is approximately 7% and 6% difference between the TP and FP, and FN and FP, respectively.

**Table 10:** Hedging and commitment.

**Term list**	**TP**	**FN**	**FP**
Epistemic adjectives	2.4	2.5	4.6
Epistemic nouns	2.3	2.2	4.0
Epistemic adverbials	2.5	2.5	4.1
Indefinite quantifiers	3.6	6.8	5.3
Modal verbs	8.3	9.5	15.0
Epistemic lexical verbs	12.7	16.3	18.2
Speculative terms [37]	13.0	14.2	16.1

Any hedging terms	25.0	32.0	37.5
Any commitment terms	40.3	48.3	36.7

Only hedging terms	15.3	15.4	25.0
Only commitment terms	30.7	31.7	24.0

Percentage of true positives, false negatives, and false positives, containing terms from the sets of hedging and commitment terms.

We have investigated the overall importance of hedging terms by combining the hedging categories into one term list (any hedging terms), and identified those TP, FN and FP which contain any of these terms (Table 10). Approximately 12% more FP contain hedging terms than TP. In contrast to hedging, we also considered if terms expressing commitment could be discriminative. As expected many of the TP and FN contain commitment terms, 40.3% and 48.3% respectively, however almost as many FP contained positive terms (36.7%) as those containing hedging terms (37.5%).

So far our analyses have not considered the possibility of both hedging and commitment terms appearing in the same sentences, as in Example 18. When we consider sentences with hedging terms and no commitment terms (only hedging terms), the FP are separated from the TP and FN almost as much as using the any hedging term, with fewer TP and FN matching.

These results indicate that hedging and commitment is common within the MIM corpus, as well as in the FP, and thus these categories cannot be used directly to detect and filter FP. However, they may be useful, in combination with other features, in the development of statistical NLP models for distinguishing between TP and FP.

## Conclusion

In this paper, we have explored the numerous advantages of, and the complexities which arise when, extracting molecular interactions from full-text articles. Our analysis is made possible through the development of our unique Molecular Interaction Map (MIM) corpus. The MIM corpus documents the manual identification of molecular interaction facts from full-text articles, which are cited in a MIM developed by Kohn [20]. Each fact can be derived from one or more passages from the citations, and each of these instances are annotated with their location in the article, and whether coreference and/or negated expressions need to be resolved for the fact to be correctly extracted. Our annotation scheme also introduces the use of factual dependencies to incorporate additional knowledge which is required to infer a fact from an instance. These dependencies model the coherency within an article as well as any assumed domain knowledge.

The MIM corpus consists of 2162 annotated sentences from 78 full-text articles. Our corpus analysis demonstrates that full-text processing is crucial for extracting biomedical knowledge. Less than 45% of individual facts and 20% of instances we identified were contained within the abstract. Our evaluation of instance dependencies also highlights the need for full-text IE. The majority of instances with dependencies, rely on statements within different article sections. Further, a full-text system would be able to exploit the redundancy of facts throughout the articles. This will increase recall, and also the likelihood of an imperfect system identifying a fact. Therefore, it is expected that systems will significantly improve by processing full-text articles.

Using our corpus we can quantify the proportion of interaction instances requiring dependencies and also the amount of external knowledge required. Only 29% of synonyms and 46% of extra facts were identified in the articles. Further our corpus allows us to report on the relative importance of NLP tools for resolving coreference and negated expressions — 13% and 5.5% of instances require at least one coreference or negated expression to be resolved, respectively, for the original MIM fact to be extracted.

We concluded our analyses with oracle sentence retrieval experiments using the MIM corpus as a gold-standard evaluation dataset. The corpus allows us to explore the characteristics of both false negative and false positive sentences. We find the majority of FN arise from extra fact dependencies and anaphoric expressions. We also investigated the use of hedging and commitment terms as a means to reduce FP.

The research presented here, provides important empirical guidance for developers of biomedical IE and IR systems. We expect systems to gain significantly by processing full-text articles instead of individual abstracts, and by incorporating coreference resolution components. Furthermore, the automatic identification of synonyms, extra facts, and mutations, will be critical for the identification of molecular interactions.

## Methods

In this section, we discuss our annotation scheme and the full-text sentence retrieval system. We provide details on the process used to construct keywords and queries, and the gold standard text used in the sentence retrieval experiments. We also present the metrics used to evaluate the system, and examples of both hedging and commitment terms.

### Annotation schema

The MIM corpus is provided in an XML format. XML is a standard markup language for structured text, and XML parsers are freely available. This section introduces the elements within the corpus.

#### Fact and instance annotation

Each fact type is marked with a specific element: mainfact, subfact, synonym, or extra. Each has an id attribute, and a fact attribute representing the specific MIM fact. The subfact elements also have a parent attribute linking it to its corresponding parent fact. Below the fact level are the instance elements. The instance elements contain the attributes: id, author and location. Any dependencies of an instance are marked within the instance element with an empty dep element. The dep element has a single attribute src which links the instance to a synonym or extra element. The example below shows another instance of the A1 Subfact in Example 11 (Figure 7).

<synonym id = "A1:s1s1" fact = "HsRad51 = Rad51">

   <instance id = "A1:s1s1.i1" author = "Yuan1998" location = "Introduction">

The finding that human Rad51 (HsRad51) promotes homologous pairing and strand exchange reactions in vitro has suggested that Rad51 may also play a role in recombinational repair in man (26).

   </instance>

</synonym >

<mainfact id = "A1:s1f1" fact = "ATM is in a complex with c-Abl and Rad51">

   <instance id = "A1:s1f1.i1" author = "Yuan1998" location = "Results">

      <dep src = "A1:s1s1"/>

In this context, HsRad51 forms a complex with c-Abl that includes ATM (data not shown).

   </instance >

</mainfact>

#### Coreference expressions

In the MIM corpus, the coreference expressions and their antecedent/s are marked as exp elements. Each exp has an id attribute which uniquely identifies the element. Coreference links are described by linking exp elements together using ptr (pointer) elements. Our scheme was adapted from Tutin *et al*. [38], which was based on the TEI's proposal for linking expressions together [39]. A pointer element specifies a relation from one point in an instance (where the ptr element appears) to one or more elements of the instance, indicated by the attribute src. For example:

In addition, Mdm2 promotes <exp id = "e.1">p53</exp> degradation, thereby terminating <exp id = "e.2"><ptr type = "anaphora" src = "e.1"/>its </exp > growth inhibitory signal.

#### Negated Expressions

The negated expression are marked with negated elements, which have a unique id attribute. For example:

<negated id = "n.1">Inactivation of p107</negated> results in the <negated id = "n.2"> loss of HDAC1 binding </negated >.

### Full-text sentence retrieval system

The sentence retrieval system takes as input queries composed of different sets of keywords which are associated with a specific fact or subfact in the MIM corpus, and retrieves all sentences matching the keywords. Each keyword search is restricted to a particular article (or articles) which is known in advance to contain the instances supporting the relevant MIM fact the query is associated with. The system does not apply any ranking criteria to the identified sentences, and thus all retrieved sentences are considered equally relevant. Our current system is not capable of searching within PDF documents, and as such the MIM corpus is slightly reduced to include only instances identified within articles in HTML format (63 full-text articles). We will refer to this reduced corpus as the MIM IR corpus, which is described later in this section.

The task of the system is to retrieve just those sentences annotated in the MIM IR corpus. Any retrieved sentence, which matches the keyword queries for a specific fact and also appears as an annotated instance of the fact is considered a relevant result, that is, a true positive (TP). Any other sentence retrieved by the system is irrelevant, and is referred to as a false positive (FP). Finally, any fact's annotated instances that are not retrieved by its corresponding queries are false negatives (FN).

### Keywords and queries

For each of the main facts and subfacts in the MIM IR corpus, we generated keyword lists containing the main terms associated with the facts and their instances. These lists were created semi-automatically by first obtaining the most frequent terms from the fact descriptions and instances, excluding any stop words, like *the *and *it*. This ensures that all main verbs associated with a fact (not only those within Kohn's description) are included. Each list was then manually reduced by a domain expert to include only those associated with the fact. These remaining terms were then divided manually into three classes: bio-entities, verbs, and auxiliary terms.

The *bio-entities *consist of all terms referring to the molecules involved, as well as their synonyms which are defined by dependencies, in the molecular interactions stated in the corresponding MIM fact. Bio-entities which occur in instances, but do not appear in the MIM fact, are excluded. The set of *verbs *includes all terms which describe the interaction relationship, as well as their synonyms. The *auxiliary *term list contains terms which were considered necessary by the domain expert to fully identify the entire MIM fact. Auxiliary terms often refer to specific structures within the bio-entities involved in the interaction, and are added manually if they are not identified by the semi-automatic approach. In many cases, no auxiliary terms are specified.

For examples, Figure 8 shows the keyword lists for the MIM A2 Subfact and N4 Main fact in Figure 6. The synonyms are shown in parentheses. Note that, the A2 keywords do not have any auxiliary terms, and the bio-entity *p70 *in the N4 instance (Example 8, Figure 6) is not included in the N4 keyword list because it isn't part of the main fact.

From these sets of keywords, five query types are specified:

**bio-ent **sentences must contain all main bio-entity terms or their synonyms

**verb **sentences must contain all main verbs associated with a MIM fact

**verb syn **as above, but sentences may contain synonyms for each main verb

**auxiliary **if a MIM fact is associated with auxiliary terms, sentences must contain these or their synonyms

**any verb **sentences must contain at least one verb from the set consisting of all verbs associated with all MIM facts in the corpus.

These five classes are then combined to construct queries with various levels of relaxation, such as ent + verb syn, which will retrieve sentences identified by both the ent and verb syn queries.

### Preparing the IR corpus

The IR experiments reported in this paper are based on 63 of the 78 full-text articles that were used to construct the MIM corpus. These articles correspond to those which are available in HTML format rather than only PDF. The HTML articles were converted into plain text using the World Wide Web browser Lynx, followed by some manual post-processing to filter out any remaining noise. Individual sentences were identified using a boundary detector based on the MXTerminator [40]. Manual post-processing was carried out to correct any mistaken boundaries, such as *et al*.. These sentences were tokenised, ensuring single term entities with punctuation, like *E2F-4*, were not split into multiple tokens.

The reduced final dataset contains 19,117 sentences and 363,130 tokens. There are 316 different facts and subfacts identified within this dataset, corresponding to 1635 individual instances and 1736 sentences annotated. Only 92 instances consist of two or more adjacent sentences, which are all required to infer their associated fact.

### Evaluation metrics

The sentence retrieval system's performance was evaluated using three metrics: precision (*P*), recall (*R*) and F-score (*F*), which are defined as follows:



where TP, FP and FN correspond to the number of true positive, false positive and false negative sentences retrieved.

### Example hedging and commitment terms

The following lists contain example terms from each hedging category and the set of commitment terms.

**Modal verbs **could, should, might

**Epistemic adjectives **probable, possible, unlikely

**Epistemic nouns **chance, claim, suggestion

**Epistemic adverbials **maybe, perhaps, presumably, surely

**Epistemic lexical verbs **appear, hypothesize, presume, suggest

**Indefinite quantifiers **about, generally, often, sometimes

**Speculative words **[37] likely, may, suggest, promise

**Commitment terms **demonstrate, established, indicating

## Availability and requirements

**Project name: **Molecular Interaction Map corpus

**Project homepage: **http://www.it.usyd.edu.au/~tara/mim_corpus/

**Operating systems: **Platform independent

## Authors' contributions

TM is the primary author of this work. TM developed the annotation scheme and annotated the MIM corpus, with contributions from JC. TM implemented the keyword extraction system, and manually edited the keyword sets. TM and JC developed the sentence retrieval experiments, and TM implemented the sentence retrieval system. The text of the paper was drafted by TM, and co-edited by JC. Both authors were involved in planning the study, and both read and approved the final manuscript.
